# Focused ultrasound neuromodulation for psychiatric disorders: a scoping review of clinical applications and current progress

**DOI:** 10.1007/s00702-026-03113-3

**Published:** 2026-02-19

**Authors:** Dillan Prasad, Rishi Jain, Bibek Samal, Nikhil Sriram, Austin Drysch, Saranya S. Menon, Ashley N. Selner, James M. Mossner, Joshua M. Rosenow

**Affiliations:** 1 Department of Neurological Surgery, Northwestern University Feinberg School of Medicine, 676 N Saint Clair Suite 2210, Chicago, IL 60611 USA; 2 Department of BioSciences, Rice University, Houston, TX USA; 3 Department of Psychiatry, University of Illinois College of Medicine, Chicago, IL USA

**Keywords:** Focused ultrasound, Functional neurosurgery, FUS, LIFU, Neuromodulation, Psychiatry

## Abstract

Low-intensity focused ultrasound (LIFU/FUS) is a precise, reversible, incisionless technology that has demonstrated potential efficacy as a neuromodulatory therapy for psychiatric disorders. While high-intensity focused ultrasound (HIFU) is routinely used in ablative neurosurgical procedures, low-intensity focused ultrasound remains investigational and is not yet ready for widespread clinical use. FUS leverages mechanisms including mechanotransduction, sonoporation, or flexoelectric effects to excite or inhibit neural tissue. In this scoping review, we synthesize the current evidence on therapeutic efficacy, safety, and sonication parameters, examining outcomes across diverse psychiatric indications. Our search identified 320 studies, of which 14 met our inclusion criteria. All studies investigated neuromodulatory FUS in subjects with psychiatric diagnoses including major depressive disorder (MDD), generalized anxiety disorder (GAD), schizophrenia, obsessive-compulsive disorder (OCD), and substance use disorder (SUD). Symptomatic improvement was reported across psychiatric indications; however, positive randomized trial results have only been demonstrated in a subset of trials, while evidence for other indications remains preliminary and is largely derived from small open-label cohorts. Effect durability was mixed. Across protocols, substantial heterogeneity exists in sonication devices, parameters, and cranial targets, suggesting a lack of consensus for technical aspects of this emerging technology. Two serious adverse events were reported. Early evidence from this emerging field suggests that FUS may improve outcomes across a variety of psychiatric indications; however, significant challenges remain, including protocol and parameter optimization, regulatory approval, and ethical considerations, emphasizing the need for continued research and interdisciplinary collaboration to optimize FUS as a therapeutic modality for patients with treatment-resistant psychiatric disorders.

**Supplementary Information:**

The online version contains supplementary material available at 10.1007/s00702-026-03113-3.

## Introduction

### History

Noninvasive brain stimulation (NIBS) technologies have been used by clinicians and researchers to modulate the brain at clinically relevant neural networks (Arulpragasam et al. 2022; Padberg et al. 2021). These technologies include transcranial magnetic stimulation (TMS), transcranial electrical stimulation (tES), electroconvulsive therapy (ECT), magnetic seizure therapy (MST), and transcranial near-infrared stimulation (tNIRS), among others (Davidson et al. 2024). While TMS and ECT have been mainstays of clinical practice for treating refractory depression, limitations including lack of data, poor focality, cost, and complexity have created a need for the development of better technologies. The emergence of low-intensity focused ultrasound (FUS) therapy, a NIBS technology which appears precise, incisionless, and reversible, offers promise for treating psychiatric disorders (Arulpragasam et al. 2022; Asher et al. 2023; Henn et al. 2024).

Neuromodulatory ultrasound traces back to 1929 when Harvey et al. discovered that ultrasonic stimulation of the frog sciatic nerve could induce gastrocnemius twitching and alter heart rate, an early sign of its ability to modulate neural tissue (Harvey 1929). Using a quartz crystal oscillator, the authors delivered continuous frequencies between 500 kHz and 3 MHz to isolated frog muscle submerged in fluid baths (Harvey 1929). This early experiment demonstrated that ultrasound could directly affect excitable tissues laying foundational knowledge for modern ultrasound devices which have replaced submerged oscillators with technology such as phased-array transducers guided by real-time MRI. Over the nearly 100 years since Harvey 1929, ultrasound has undergone numerous phases of development, application, and dormancy; in 1939, therapeutic ultrasound in humans was first successfully used for the treatment of painful neuralgia; in 1942, the first diagnostic ultrasonic image of the brain was produced (Beisteiner 2022; Darrow 2019; Dussik 1942; Franzini et al. 2020; Van Tiggelen and Pouders 2003). The resurgence of the neuromodulatory potential of ultrasound in the modern era has been attributed to Hameroff et al., who reported in 2013 that unfocused transcranial ultrasound durably improved subjective mood and global affect in patients with chronic pain, prompting renewed investigation into ultrasound for psychiatric indications (Beisteiner 2022; Hameroff et al. 2013).

Transcranial ultrasound can be unfocused (TUS) or focused (FUS) based on the presence of an acoustic lens to concentrate sound waves and direct energy to a specific location (Hospital 2025). TUS is unconcentrated and exposes a broad brain region to sonication; by contrast, FUS confines energy to a small focal zone, usually under MRI guidance, hence the term magnetic resonance-guided focused ultrasound (MRgFUS). This achieves high spatial precision within deep or superficial brain structures. FUS may be termed high-intensity (HIFU) or low-intensity (LIFU) depending on the amount of power per unit area delivered to the target location (Bachu et al. 2021). HIFU can produce focal thermal effects inducing cell death and is routinely used in a variety of neurosurgical procedures for conditions such as essential tremor, tremor-dominant Parkinson’s disease, and Parkinson’s dyskinesias, which are FDA-approved indications for MRgFUS. Indications under active investigation include focal refractory epilepsy (e.g. periventricular nodules, hypothalamic hamartoma), anterior capsulotomy for treatment-resistant obsessive compulsive disorder (OCD), and others (Beisteiner et al. 2023; Bretsztajn and Gedroyc 2018; Jung and Chang 2018; Meng et al. 2021). LIFU is delivered at far lower intensity, is non-thermal, and produces reversible effects, such as disruption of the blood-brain barrier and modulating neural function (Arulpragasam et al. 2022; Beisteiner et al. 2023; Carpentier et al. 2024; Tu and Yu 2022). HIFU and LIFU are both generally performed under real time MRI guidance, though the term MRgFUS typically describes HIFU, not LIFU (Henn et al. 2024).

Both HIFU and LIFU have garnered significant interest for their potential to treat psychiatric illness. Other groups have reviewed HIFU in the context of psychiatric neurosurgery, concluding that current applications largely focus on OCD and treatment-resistant depression (Henn et al. 2024). Several recent reviews have comprehensively summarized neuromodulatory transcranial ultrasound (TUS/FUS/LIFU), including psychiatry-focused syntheses, broader clinical overviews spanning neurological and psychiatric indications, systematic reviews integrating preclinical and clinical evidence with attention to neural readouts including neuroimaging and key methodological confounds, and parameter-effect relationship analyses intended to guide protocol optimization (Keihani et al. 2024; Matt et al. 2024; Pellow et al. 2024; Nandi et al. 2024). In parallel, international consensus efforts such as ITRUSST have proposed standardized reporting frameworks and practical best-practice guidance to improve safety, reproducibility, and cross-study comparability (Martin et al. 2024; Murphy et al. 2025). In contrast, our aim is explicitly clinical and translational. Here, we restrict our synthesis to human studies enrolling participants with diagnosed psychiatric disorders, emphasizing effect durability, pragmatic protocol choice, and what remains unknown for physicians considering early adoption of this technology.

### Mechanisms

Focused ultrasound delivers ultrasonic pulses from an external transducer to an intracranial target. Several mechanisms have been proposed to link the vibrations generated by ultrasonic waves to neural tissue activation, though research is ongoing and consensus as to the definite mechanism has not yet been reached (Blackmore et al. 2019). Potential mechanisms include mechanotransduction of ion channels, flexoelectric or cavitation-related effects through membrane perturbation, sonoporation causing alterations in membrane ion permeability, or combinations of these (Tu and Yu 2022; Blackmore et al. 2019; Coste et al. 2010; Gottlieb and Sachs 2012; Wu et al. 2016; Yoo et al. 2022; Oh et al. 2019; Sorum et al. 2021; Lengyel et al. 2021; Qiu et al. 2019; Zhu et al. 2023; Jerusalem et al. 2019; Krasovitski et al. 2011). The resulting effect can either excite or inhibit neural tissue depending on the neuroanatomic target and sonication parameters (Blackmore et al. 2019).

Piezoreceptors are mechanosensitive cell membrane ion channels that transduce mechanical forces such as pressure, stretch, and shear stress into electrochemical signals, playing a critical role in cellular transduction (Coste et al. 2010; Gottlieb and Sachs 2012; Wu et al. 2016). These ion channels are non-selective cation channels primarily permitting the influx of calcium (Ca²⁺), as well as sodium (Na⁺) and potassium (K⁺) upon activation (Coste et al. 2010; Gottlieb and Sachs 2012). In neural tissue, piezoreceptor activation results in an initial Ca²⁺ influx, which subsequently triggers intracellular signaling cascades that amplify neuronal excitation through calcium and voltage-gated channels, ultimately generating burst firing responses (Yoo et al. 2022). Mechanosensitive ion channels implicated in ultrasound neuromodulation extend beyond piezoreceptors, encompassing transient receptor potential (TRP) channels such as TRPA1, TRPP1/2, TRPC1, and TRPV1, as well as potassium channels like TREK1 and TRAAK, and voltage-gated calcium channels (VGCCs) (Yoo et al. 2022; Oh et al. 2019; Sorum et al. 2021; Lengyel et al. 2021).

Among the piezoreceptor family, Piezo1 has been implicated in mediating the effects of ultrasonic neuromodulation. Expressing mouse Piezo1 in otherwise insensitive HEK-293T cells has been shown to be sufficient to confer robust, pressure-dependent Ca^2+^ influx under sonication, supporting its role in force detection (Qiu et al. 2019). In vivo, conditional knockout of Piezo1 in mouse motor cortex neurons markedly reduces ultrasound-induced cortical calcium signals, contralateral limb movements, EMG amplitudes, and c-Fos expression (Zhu et al. 2023). The same study maps Piezo1 across the cortex, central amygdala, hypothalamus, Edinger-Westphal nucleus, and red nucleus, while other work demonstrates its presence in peripheral mechanosensory neurons and vascular endothelium (Coste et al. 2010). Together, these data support that Piezo1 may be both necessary and sufficient to translate acoustic forces into neuronal activation.

Other hypothesized mechanisms which may underlie ultrasonic neuromodulation include flexoelectricity (the induction of electric polarization by a gradient of mechanical strain), sonoporation, direct membrane disruption allowing for increased ion permeability, and cavitation effect, in which pockets of transient microbubbles are created within the cellular membrane bilayer resulting in cellular capacitance changes (Tu and Yu 2022; Blackmore et al. 2019; Jerusalem et al. 2019).

Current evidence supports that LIFU can produce either excitatory or inhibitory neuromodulation depending on which membrane channels are recruited and how sonication is timed. Piezo1-mediated calcium influx and neuronal depolarization have been demonstrated in vitro by Qiu et al. and in vivo by Zhu et al., while other groups have showed that focused ultrasound excites cortical neurons via mechanosensitive calcium accumulation and ion channel amplification (Yoo et al. 2022; Qiu et al. 2019; Zhu et al. 2023). Additional literature highlights that astrocytic TRPA1 activation under pulsed ultrasound can drive glutamate release and subsequent neuronal spiking, providing another excitatory pathway (Oh et al. 2019). In contrast, inhibitory effects have been attributed to activation of two-pore domain potassium (K2P) channels, specifically TRAAK, which opens in response to ultrasound-induced membrane tension and hyperpolarizes neurons, hence reducing firing probability (Sorum et al. 2021). Mechanistic reviews of ultrasound neuromodulation further emphasize that the net polarity of these effects is parameter dependent. Shorter, low-duty-cycle pulses tend to favor transient excitation, while longer pulse trains or higher duty cycles are more likely to cause net suppression through cumulative hyperpolarizing currents or network-level dampening (Blackmore et al. 2019; Kamimura et al. 2020). However, further translational and human data are required to establish under which conditions LIFU reliably produces excitatory or inhibitory effects and to better define how stimulation parameters interact with individual and population-level cell signaling.

### Parameters

Sonication protocols are highly customizable based on device and parameter choice. Manipulating parameters can induce excitatory or inhibitory effects (Blackmore et al. 2019). The parameter space includes the fundamental frequency (which balances focal precision with skull penetration), the pulse regime (pulse duration, PD; and pulse repetition frequency, PRF) that together define the duty-cycle and bias the bioeffect toward mechanical or thermal, and two intensity metrics—spatial‑peak pulse‑average intensity (I_SPPA_) and spatial‑peak temporal‑average intensity (I_SPTA_)—that respectively bound instantaneous mechanical stress and cumulative heating (Naor et al. 2016). The mechanical index (MI) provides an additional cavitation-safety margin; the FDA specifies that MI <1.9, I_SPPA_ <190 W cm⁻², and I_SPTA_ <720 mW cm⁻² to ensure negligible temperature rise in the context of neuromodulation (Blackmore et al. 2019; Marketing 2023). Typical neuromodulatory protocols therefore operate at far lower intensities than ablative exposures (Table 1).

**Table 1:** Description of ultrasound parameters

Parameter	Description	Significance
Frequency	Central carrier frequency of the acoustic wave. Typical human neural stimulation range: 220 - 700 kHz.	- Lower-kHz beams experience less skull attenuation and therefore reach deeper targets - Higher frequencies give smaller focal spots but lose energy faster through bone
Pulse Duration (PD)	Time each burst is “on” in µs-ms.	- Short PD minimizes heating and favors mechanical (ion channel) effects
Pulse Repetition Frequency (PRF)	Rate at which ultrasound pulses are emitted per second (Hz).	- Higher PRF → increased pulse delivery, raising total energy - Biases towards net excitation versus inhibition
Duty Cycle (DC)	Percentage of each PRF period that the transducer is emitting. DC = PD x PRF	- Higher DC → increased heating effects - Typical neuromodulatory DC <5% before heating starts
Spatial-Peak Pulse Average (I_SPPA_)	Average intensity of the ultrasound pulse during the active “ON” portion of the pulse only (W/cm^2^)	- High I_SPPA_ → increased mechanical effects during sonication - Per FDA, ISPPA < 190 W/cm^2^
Spatial-Peak Temporal Average (I_SPTA_)	Average intensity over an entire pulse cycle (mW/cm^2^). I_SPTA_ = ISPPA × DC	- High ISPTA → increased thermal effects during sonication - Determines safe ultrasound neuromodulation levels - Per FDA, ISPTA < 720 mW/cm^2^
Mechanical Index (MI)	Measures the likelihood/exposure of mechanical effects such as inertial cavitation. MI = Peak pressure / √(Center frequency)	- Higher MI → increased risk of tissue damage due to cavitational stress. - Per FDA, MI < 1.9.

This table provides an overview of key ultrasound parameters relevant to neuromodulation. Each parameter influences either spatial targeting, energy deposition, or safety considerations.

### Targets

**Fig. 1:**
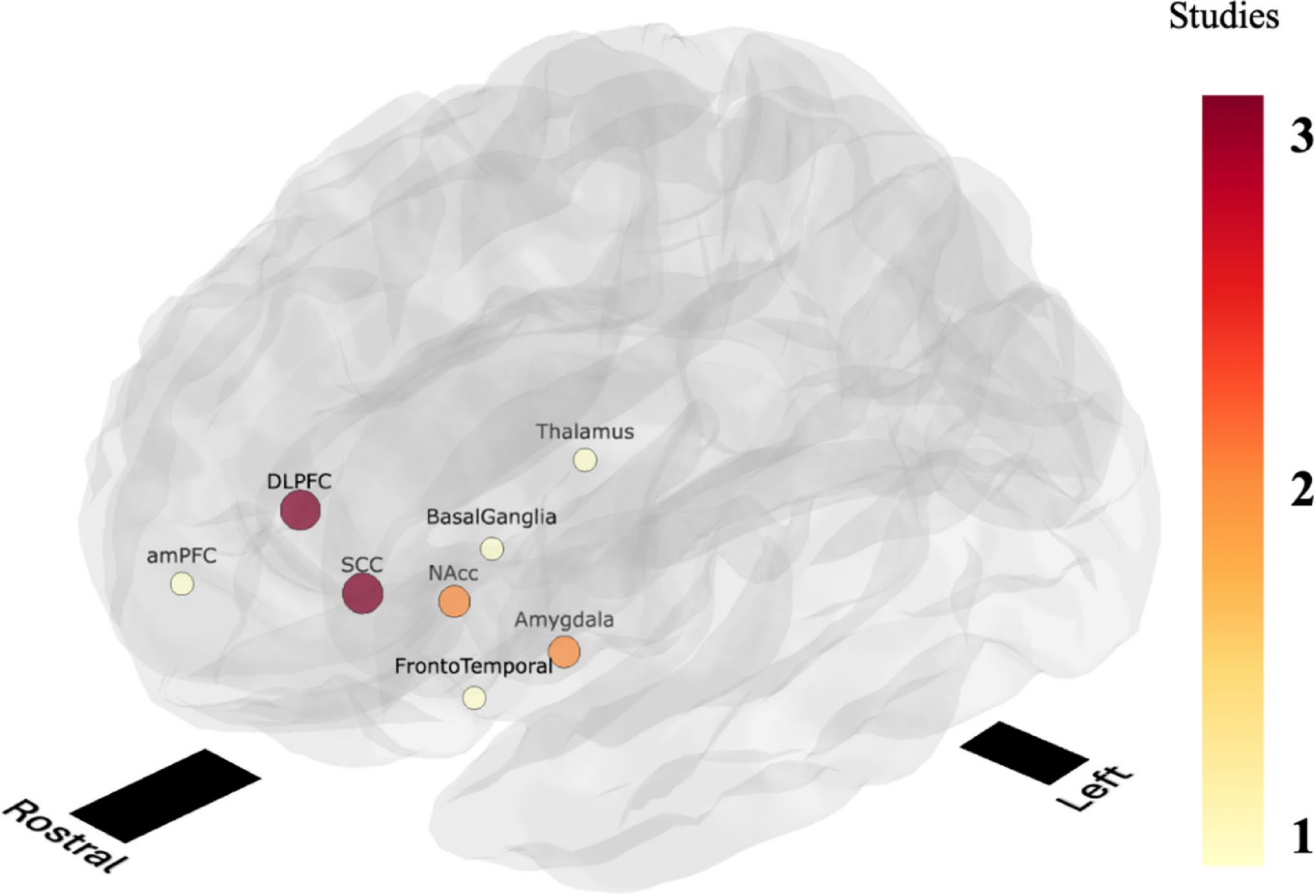
Neural targets of selected focused ultrasound neuromodulation studies for psychiatric indications

In early human applications, LIFU has primarily been directed at regions with established roles in affective and cognitive circuitry. Targets such as the amygdala and subgenual anterior cingulate cortex (sgACC) have been selected based on their central involvement in mood regulation, emotional salience processing, and network-level dysfunction in depression and anxiety disorders (Langevin et al. 2016; Mayberg et al. 2005). The dorsolateral prefrontal cortex (DLPFC) remains a critical hub within cognitive control signaling and has served as the principal cortical target for noninvasive neuromodulation modalities such as TMS and TBS (Miron et al. 2021). In disorders characterized by reward, motivation, or compulsive behavior, the nucleus accumbens and ventral striatum are recurrent targets given their roles in valuation and action selection circuitry and their established relevance in both DBS and focused neuromodulation (Figee et al. 2013). A study-level summary alongside corresponding brain structure targets used in included LIFU psychiatric investigations is provided in Table 2 and visually highlighted in Figure 1.

## Methods

### Scoping review

A scoping review methodology was chosen to map and critically appraise the current evidence on neuromodulatory ultrasound for psychiatric disorders, with emphasis on how stimulation mechanisms, experimental design, parameter settings, and neuroanatomical targets influence therapeutic outcomes. As the technology is relatively nascent and consensus on optimal parameters, targets, and indications has not yet been reached, a scoping review offers an appropriate framework to survey available findings and identify trends. Insights from this synthesis are intended to guide the development of standardized experimental paradigms and clinical protocols for future investigations of ultrasound neuromodulation.

**Table 2:** Studies examining focused ultrasound neuromodulation in human patients with psychiatric illness.

Author and Year	Title	Design/Subjects	Targets	Sonication parameters	Indexes	Key findings	Serious adverse events	Clinical Indications	Ultrasound Device
Reznik 2020	A double-blind pilot study of transcranial ultrasound (TUS) as a five-day intervention: TUS mitigates worry among depressed participants	Mild-to-moderate depression, n = 24, double-blind pilot study	Right fronto-temporal cortex	FF = 500 kHz Duration = 30 s (single train) ISPTA = 71 mW cm⁻² ISPPA = 14 W cm⁻² MI = 0.92 Peak -p ≈ 0.65 MPa 30 s per session; 5 sessions	BDI-II, OASIS, PSWQ, VAMS	Active TUS produced a small, short-term drop in trait worry, while worry rose in sham; depressive and anxiety severity were unchanged, and no group differences persisted at 1-month follow-up.	No	MDD	Neurotrek U+
Riis 2023	Durable effects of deep brain ultrasonic neuromodulation on major depression: a case report	MDD patient, n = 1, case report	Subcallosal cingulate cortex	FF = 650 kHz DC = 0.8% PD = 30 ms Peak p = 1 MPa 64 min total active	HDRS-6, fMRI BOLD	SCC BOLD signal fell immediately during sonication; HDRS-6 dropped from 11 to 0 within 24 h and remission persisted 44 days. Patient described a “brain woken up” feeling.	No	MDD	Custom/unknown (research prototype)
Cheung 2023	Effects of Transcranial Pulse Stimulation (TPS) on Adults with Symptoms of Depression-A Pilot Randomized Controlled Trial.	MDD patients, n = 30, single-blinded, parallel-group RCT	DLPFC	Pulse duration: ~ 3 µs Energy flux density: 0.2-0.25 mJ/mm⁻² Pulse repetition frequency: 3-4 Hz 300 pulses per session, six 30-minute sessions	HDRS-17, SHAPS, MoCA, Digit Span-Backward, IADL, TMT-A/B	Immediate mood lift after six MRI-guided TPS sessions to the left DLPFC that deepened over three months, accompanied by marked relief of anhedonia and sizeable gains in global cognition and working-memory.	No	MDD	NEUROLITH TPS generator, Storz Medical AG
Fan 2024	Thalamic transcranial ultrasound stimulation in treatment resistant depression	MDD patient, n = 1, case report	ANT, VC, BNST	FF = 500 kHz PRF = 25 Hz DC = 13% Pulse train duration = 300 s ISPPA 42-50 W cm⁻² 8 trains total (4 left, 4 right) alternating sides	VAS-D, HAMD-6, rs-fMRI	ANT stimulation produced acute VAS-D reduction and DMN connectivity normalization; HAMD-6 trended downward but was not significant. VC/BNST and unfocused control showed smaller or no effects.	No	MDD	Attune ATTN201 wearable device
Oh 2024	Effect of Low-Intensity Transcranial Focused Ultrasound Stimulation in Patients With Major Depressive Disorder: A Randomized, Double-Blind, Sham-Controlled Clinical Trial	MDD patients, n = 26, double-blind, sham-controlled RCT	Left DLPFC	FF = 250 kHz ISPPA = 3 W cm⁻² Peak -p = 300 kPa Tone burst = 1 ms (at DC = 50% within 300 ms sonication trains every 6 s for 20 min/session) 20 minutes per session, with 6 sessions over two weeks	MADRS, QIDS-SR, SSI, STAI, K-POMS, CANTAB, rs-fMRI	Marked mood improvement and reduced suicidal ideation after six tFUS sessions to the left DLPFC, with just over half of treated patients achieving clinical response two weeks later. rs-fMRI showed stronger connectivity between the subgenual ACC and fronto-striatal hubs only in the active arm, but these network shifts were not tied to symptom relief.	No	MDD	NS-US100; Neurosona Co. Ltd
Schachtner 2025	Transcranial Focused Ultrasound Targeting the Default Mode Network for the Treatment of Depression	MDD patients, n = 20, open-label case series	Antero-medial PFC	FF = 400 kHz PD = 5 ms PRF = 10 Hz Duty = 5 % ISPTA = 670 mW cm⁻² 10 min per session; up to 11 sessions	BDI-II, HDRS-17, PTQ, WHOQOL-BREF	Mood improved early and continued to improve throughout the 3-week course, with most patients reaching clinical response; repetitive negative thinking and physical/psychological quality-of-life scores improved. Effects were documented only up to 24 h after the last session, so long-term durability remains unclear.	No	MDD	Openwater Neuromodulation device
Riis 2025	Noninvasive Modulation of the Subcallosal Cingulate and Depression With Focused Ultrasonic Waves	MDD or BPD (current episode: depressive) patients, n = 22, randomized, double-blind sham-controlled crossover pilot	Subcallosal cingulate cortex	FF = 650 kHz Burst duration = 30 ms PD = 5 ms Peak -p = 1 MPa ISPPA = 31 W cm⁻² ISPTA = <720 mW cm⁻²; MI < 1.9 fMRI: 5 x 1-min sonications, total active 5 min Treatment: 39-41 min total	HDRS-6, PANAS-X sadness, BOLD fMRI	Target-specific SCC BOLD deactivation during stimulation. In per-protocol group, real > sham for PANAS-X Sadness change immediately post-treatment and HDRS-6 at 24 h; no significant difference at 7 days (per-protocol). Intent-to-treat showed nonsignificant trends.	Yes	MDD, BPD	Custom/unknown (research prototype)
Barksdale 2025	Low-intensity transcranial focused ultrasound amygdalaneuromodulation: a double-blind sham-controlled target engagement study and unblinded single-arm clinical trial	Mood, anxiety, and trauma-related disorders (n = 29), double-blind sham-controlled target engagement study; unblinded single-arm clinical trial	Left amygdala	PRF = 10 Hz PD = 5 ms DC = 5 % ISPPA = 14.4 W cm⁻² ISPTA ≈ 720 mW cm⁻² Peak -p = 0.64 MPa 10 min per visit; 15 visits	MASQ-General Distress (GD), fMRI BOLD, numerous others	Repeated amygdala-focused ultrasound dampened amygdala activity and over 3 weeks eased depressive, distressive, anxiety, and PTSD-related symptoms, though numerous side effects were reported.	No	MDD, GAD, PTSD	Brainsonix Pulsar 1002 device
Attali 2025	Deep transcranial ultrasound stimulation using personalized acoustic metamaterials improves treatment-resistant depression in humans	MDD patients, n = 5, open-label pilot	Subcallosal cingulate cortex	FF = 500 kHz PD = 4.5 ms PRF = 14 Hz Overall DC = 1.8% ISPTA ≈ 184 mW cm⁻² 5 min sessions; 5 sessions/day; 5 days	MADRS, HDRS-17/-6, QIDS-SR, neuropsych tests, rs-fMRI	Metalens (custom acoustic disc accounting for patient-specific skull differences) system trialed. Five-day protocol produced rapid, large symptom relief (mean 61% MADRS improvement, 4/5 responders) though benefits substantially abated within 4-5 weeks post-sonication. Connectivity of SCC with left DLPFC increased.	No	MDD	Custom/unknown (research prototype)
Mahdavi 2023	A pilot study of low-intensity focused ultrasound for treatment-resistant generalized anxiety disorder	GAD patients, n = 25, open-label pilot	Right amygdala	FF = 650 kHz PD = 5 ms pulse PRF = 10 Hz DC = 5% MI = 0.75 Peak -p = 0.61 MPa ISPPA 14.4 W cm⁻² ISPTA 720 mW cm⁻² 10 min per visit; 8 visits	HAM-A, BAI, PGI-I	Anxiety dropped on both assessments with most patients responding to treatment and rating meaningful improvement and some patients experiencing remission of symptoms.	No	GAD	Brainsonix Pulsar 1002 device
Zhai 2023	The efficacy of low-intensity transcranial ultrasound stimulation on negative symptoms in schizophrenia: A double-blind, randomized sham-controlled study	Schizophrenia patients, n = 26, double-blind, randomized sham-controlled study	DLPFC	FF = 500 kHz SD = 500 ms ISPPA = 8.1 W cm⁻² ISPTA = 0.40 W cm⁻² 15 visits	SANS, PANSS, C-BCT (TMT-A, Symbol Coding, CPT, Digit Span)	Striking drop in negative symptoms, with SANS scores falling steeply in the active arm while sham patients were unchanged. Overall illness severity on the PANSS improved in parallel, and attention/vigilance on the Continuous Performance Test edged upward, whereas other cognitive domains remained unchanged. Correspondence (preliminary data).	No	Schizophrenia	Immersion-type focused transducer V391-SU (Olympus), custom driver
Mahoney 2023	Low-intensity focused ultrasound targeting the nucleus accumbens as a potential treatment for substance use disorder: safety and feasibility clinical trial	Opioid use disorder outpatient patients, n = 4, open-label clinical trial	Bilateral nucleus accumbens	FF: 220 kHz. PD: 100 ms ISPPA: 60 W for lower dose; 90 W for enhanced dose SD: 10 minutes per hemisphere Duty Cycle: 3.3%	Cue-induced VAS cravings, 7-day EMA, 90-day cue-reactivity test, HAM-D, CSSRS, MRI	For participants with the “enhanced” FUS protocol, cue-induced craving of substances significantly decreased and daily cravings metric also reduced in the week after treatment. Similarly, cue-induced cravings were shown to be decreased over the 90 day post-LIFU follow-up. Low dose protocol produced minimal acute effect but showed smaller long-term drops.	No	SUD	ExAblate Neuro Type 2, Insightec
Rezai 2025	Focused Ultrasound Neuromodulation: Exploring a Novel Treatment for Severe Opioid Use Disorder	Opioid use disorder patients, n = 8, prospective, open-label single-arm trial	Bilateral nucleus accumbens	FF = 220 kHz PD = 100 ms PRF ≈ 0.33 Hz DC ≈ 3.3 % 4 x 5 min blocks (20 min total)	HDRS, CSSRS, SHPS	FUS produced a 91% median opioid-craving reduction both immediately and sustained at 90 d; similar results for methamphetamine, cocaine. 7/8 patients remained abstinent at 30 d, and 5/8 at 90 d confirmed by urine toxicology. rs-fMRI showed decreased NAcc-vmPFC/ACC/PCC connectivity.	No	SUD	ExAblate Neuro Type 2, Insightec
Jordan 2023	A pilot study of transcranial low-intensity focused ultrasound for treatment-resistant obsessive-compulsive disorder	Treatment resistant OCD patients, n = 21, open-label pilot study	Basal ganglia (caudate nucleus; ventral striatum)	N/A 10 min per visit; 8 visits	Yale-Brown Obsessive-Compulsive Scale (Y-BOCS) score, Global Rating of Change (GRC)	First 6 patients underwent caudate nucleus targeting, next 15 underwent ventral striatum targeting, 1 patient was excluded. Among 10 completing ventral striatum targeted FUS, 70% achieved clinical response; high drop-out in caudate arm (2 patients completed treatment). Results suggest the ventral striatum is a more tolerable and possibly efficacious target. Research abstract (preliminary data).	No	OCD	Unknown

This table summarizes key findings from various studies investigating neuromodulatory FUS in psychiatric conditions. The table includes details on study authors and publication year, study design and subject characteristics, targeted brain regions, ultrasound sonication parameters, outcome measures, key findings, modulatory effects, adverse events, clinical indications, and the ultrasound device used.

### Search strategy and criteria

This scoping review was conducted following the PRISMA-ScR guidelines (Tricco et al. 2018). Comprehensive searches were performed across three major databases: PubMed, Embase, and Scopus. The search strategy aimed to capture relevant literature on ultrasound neuromodulation for psychiatric disorders. Full search terms can be found in Supplementary Table 1. The search, conducted in May of 2025, included primary research on neuromodulatory ultrasound interventions in patients with diagnosed psychiatric disorders ranging in severity. The search results were imported into a reference management system and duplicates were removed. Two independent reviewers (BS and RJ) screened the titles and abstracts to identify relevant studies. Articles were then assessed for eligibility based on predefined inclusion and exclusion criteria which can be found in Supplementary Table 2. Briefly, eligible studies specifically investigated the use of neuromodulatory focused ultrasound for psychiatric disorders in human subjects with definite diagnoses, reported clear therapeutic or neuromodulatory outcomes (e.g., efficacy), and included both full texts and abstracts with sufficient efficacy data. Any discrepancies in study selection were resolved through discussion or adjudicated by a third reviewer (DP) when necessary.

### Data extraction and synthesis

Four reviewers (DP, BS, RJ, NS) independently extracted data including study design, participant characteristics, ultrasound parameters such as frequency and intensity, targeted psychiatric conditions, neuromodulatory mechanisms, outcomes, and any reported adverse effects. The extracted data were synthesized narratively to identify patterns and trends in the application of focused ultrasound for psychiatric disorders. Given that LIFU protocols in psychiatry are typically reported in a disorder-specific manner, with noted overlap as relevant, results are discussed by diagnostic category and target-level details are provided for each study in Table 2, alongside treatment efficacy, and safety considerations. Key ultrasound parameters were further summarized to highlight their implications for therapeutic outcomes.

## Results

### Literature review

**Fig. 2:**
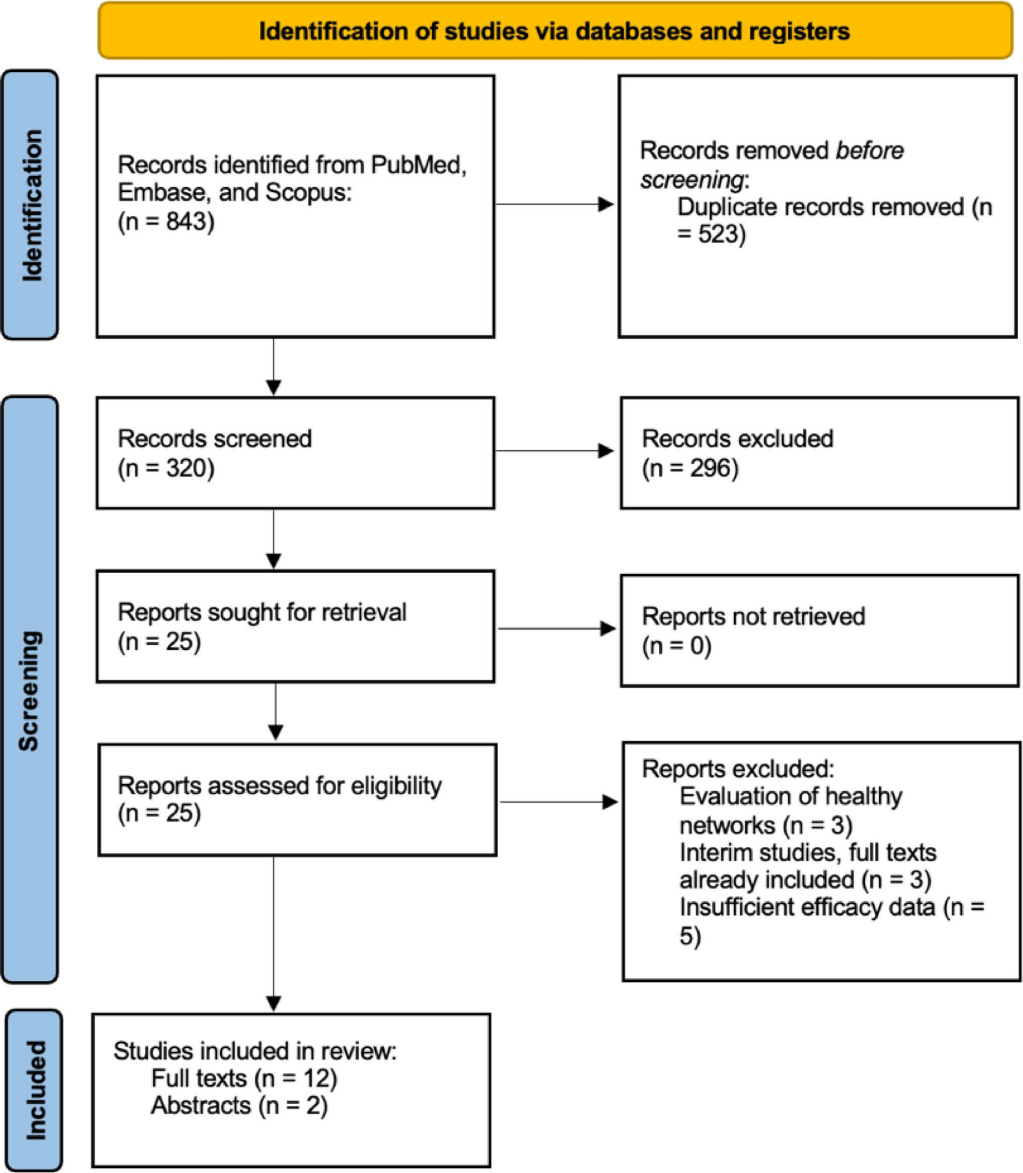
PRISMA flowchart for identification and screening of psychiatric FUS studies. This figure outlines the study selection process for the scoping review. A total of 843 records were identified from PubMed, Embase, and Scopus. After removing 523 duplicate records, 320 studies were screened based on title and abstract. Of these, 296 were excluded, and 25 full-text reports were sought for eligibility assessment. Following exclusions based on study focus (evaluation of healthy networks, interim reports, and insufficient efficacy data), 14 studies were included in the final review (12 full texts and 2 abstracts)

**Fig. 3:**
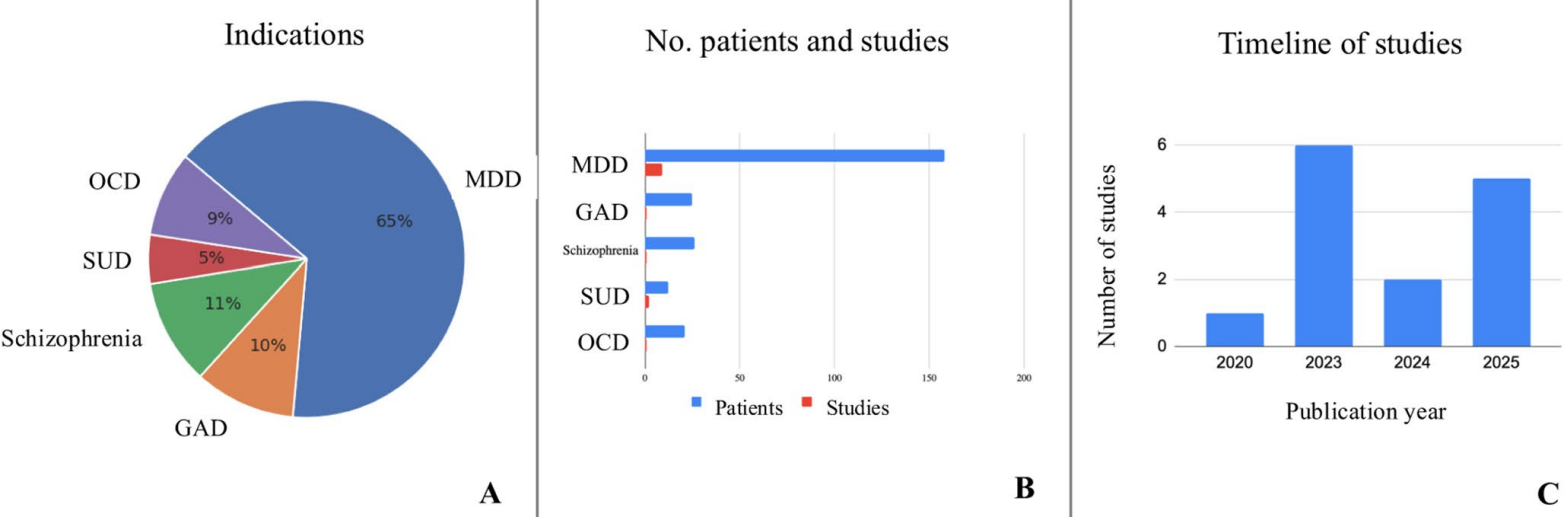
Characterization of focused ultrasound neuromodulation studies. **A **shows the distribution of focused ultrasound neuromodulation studies by patients per indication. **B **Shows the number of patients and studies by indication. **C** shows the number of studies by year

According to PRISMA guidelines, our search produced 320 unique abstracts and full-text articles, of which 14 completely fulfilled the inclusion criteria (Figure 2). Articles were published between 2020 and 2025, were mostly from the United States, and examined one of five psychiatric indications (Figure 3). Of the 14 relevant articles identified (Table 2), 9 studies focused on major depressive disorder (MDD), 1 on generalized anxiety disorder (GAD), 1 on obsessive-compulsive disorder (OCD), 2 on substance use disorder (SUD), and 1 on schizophrenia (Figure 3A). We ultimately grouped three mixed-cohort studies with MDD as a primary indication given the researchers’ choice of clinical indices; one study enrolled patients with mixed affective disorders including MDD, GAD, and trauma-related disorders; one study included subjects with “mild-to-moderate depression” and excluded severely depressed or suicidal patients; and one study consisted of a 22-patient cohort of whom 20 were diagnosed with MDD and 2 with bipolar disorder, current episode depressive (Barksdale et al. 2025; Reznik et al. 2020; Riis et al. 2025)

In total, 242 subjects were identified across the included studies, the majority of which (65%) were patients with MDD (Figure 3B). Targets of sonication varied substantially and included the amygdala, subcallosal cingulate cortex (SCC), dorsolateral and/or anteromedial prefrontal cortex (PFC), fronto-temporal cortex, thalamus, and the basal ganglia (nucleus accumbens, caudate, ventral striatum) (Figure 1). Furthermore, there was significant heterogeneity in sonication devices and protocols, assessment/follow-up timelines, and study design; for example, studies varied from open-label pilots with custom ultrasound prototype devices, to sham-controlled RCTs with commercially available devices and standardized parameters. Many studies included mild adverse events (AEs), and one study reported two serious adverse events.

### Major depressive disorder (MDD)

Nine studies investigating the effects of focused ultrasound for major depressive disorder (MDD) in 158 total patients were included in this review (Barksdale et al. 2025; Reznik et al. 2020; Cheung et al. 2023; Oh et al. 2024; Schachtner et al. 2025; Riis et al. 2025; Fan et al. 2024; Attali et al. 2025; Riis et al. 2023). Most studies reported reductions in depressive symptom severity with varying effect sizes, though outcomes were heterogeneous across study designs and outcome domains. Targets included the DLPFC, fronto-temporal cortex, SCC, amygdala, subcallosal cingulate white matter tracts, and anterior nucleus of the thalamus (ANT). Sonication parameters varied widely across the studies. Frequencies ranged from 250 kHz to 650 kHz and sonication duration ranged between 30 s and 64 min. Common outcome metrics included the Hamilton Depression Rating Scale (HDRS), Montgomery-Åsberg Depression Rating Scale (MADRS), Beck Depression Inventory-II (BDI-II), and others.

Three randomized controlled trials (RCT) were conducted for MDD two of which were sham-controlled and one waitlist controlled; a fourth study was a randomized controlled crossover pilot. Reznik et al. 2020 conducted the first placebo-controlled RCT of low-intensity ultrasound for major depression, enrolling 24 university students with mild-to-moderate symptoms (Reznik et al. 2020). Participants were randomized to active or sham groups and received five sessions of 30-second sonications of the right fronto-temporal cortex over seven days (500 kHz, I_SPTA_ ≈ 71 mW cm⁻²; Neurotrek U+, Thync, Los Gatos, CA). Given the pilot nature and small sample size, the study was underpowered for conventional between-group effects. Across the treatment week, trait worry (a dispositional tendency to engage in uncontrollable worrying, typically indexed by the Penn State Worry Questionnaire) fell in the active group and rose in sham (PSWQ, *t*(20) = −1.74, *p*= 0.097), whereas BDI-II and anxiety impairment (OASIS) scores showed no differential change. At one month follow-up, no persistent group differences in BDI-II or OASIS were detected, and trait worry was not reassessed. No adverse events occurred (Reznik et al. 2020).

The largest study reported thus far Cheung et al. reported a single-blinded RCT in 2023 with 30 MDD patients, allocating 15 to transcranial pulse stimulation (TPS) and 15 to a waitlist control. TPS is a form of low-intensity focused ultrasound that delivers single, ultrashort acoustic pulses (Cheung et al. 2023). Using MRI-guided neuronavigation, six 30-minute sessions of 300 pulses per session (pulse repetition frequency 3–4 Hz) were administered to the left DLPFC over two weeks. Compared with control, the TPS group exhibited a significant immediate reduction in HDRS-17 scores with a mean difference of −6.60 compared to the control group (*p* = 0.02, Cohen’s d = −0.93), with effects further deepening at the 3-month follow-up (Cohen’s d = −1.35) (Cheung et al. 2023). Immediate improvements were also noted in the secondary outcomes of anhedonia (Cohen’s d = −0.79), cognition (Cohen’s d = 0.88), and working memory (Cohen’s d = 0.69), each of which similarly sustained or became even more prominent at the 3 month mark (Cohen’s d = −0.81, 1.20, 1.09, respectively) (Cheung et al. 2023).

The third MDD RCT was the 2024 study reported by Oh et al. This double-blind, sham-controlled trial randomized 26 adults with MDD to real or sham ultrasound treatment; 23 completed all procedures (treatment = 11, sham = 12) (Oh et al. 2024). Each participant received six 20-min sessions of low-intensity ultrasound (250 kHz, 3 W cm^−2^ I_SPPA_, 1 ms bursts at 50% duty cycle, 300 ms trains every 6 s) to the left DLPFC over two weeks. Mean MADRS ratings fell from 28.5 ± 8.4 to 16.8 ± 6.8 at end-of-treatment, further falling to 14.8 ± 7.2 two weeks later (*p*= 0.003), whereas the sham group showed only modest change (baseline: 29.2 ± 8.3, treatment: 25.7 ± 9.0, two weeks: 24.8 ± 9.3). Response (≥ 50% reduction) occurred in 54.5% of treatment patients versus 8.3% in sham, and remission (MADRS < 9) in 18.2% versus 8%. Suicidal ideation and overall mood state also improved in the treatment arm. Resting-state fMRI analysis was obtained at baseline and within 24 h of the final session. Functional connectivity (FC; temporal correlation of spontaneous BOLD fluctuations) increased between the right subgenual/subcallosal ACC (BA 25) and four regions (left medial PFC, left middle frontal gyrus, right caudate, left orbitofrontal cortex) only in the treatment group (Oh et al. 2024). Prior studies have suggested that hypoactivation and decreased volume of the sgACC may be related to symptomatology of major depressive disorder (Rodríguez-Cano et al. 2014). However, FC between the stimulated DLPFC and the ACC did not change, and FC shifts were not linked to clinical improvement. No imaging or longer follow-up was reported; further durability of effects was not assessed (Oh et al. 2024).

Schachtner et al. 2025 conducted an open-label case series targeting the default-mode network (DMN) in 20 adults with MDD who also scored high on repetitive negative thought, a state thought to be associated with DMN hyperconnectivity in depression (Schachtner et al. 2025). Participants received up to 11 ten-minute sessions of low-intensity ultrasound over one to three weeks (five sessions in week 1, then three sessions per week for two additional weeks if early-remission criteria were not met). Sonications (400 kHz, 5 ms pulses at 10 Hz, I_SPTA_ ≈ 670 mW cm⁻²) were targeted to the anterior medial prefrontal cortex (amPFC), a default-mode network hub. Changes were measured at baseline, again after one week, then again upon conclusion of the final session; by end of treatment, BDI-II scores fell by 10.9 points (*p* < 0.001) while HDRS-17 fell by 4.2 points (*p* < 0.001). Response rates were 60% (BDI-II) and 45% (HDRS); 35% met remission on both scales. Repetitive negative thought (PTQ) scores dropped 8.4 points (*p* < 0.001) and the magnitude of PTQ change predicted symptom improvement (R² = 0.67 for BDI-II change). Overall quality of life improved as assessed by physical, psychological, and environmental subscores of WHOQOL-BREF (*p *≤ 0.001); social satisfaction was unchanged. No serious adverse events were detected. No assessments were performed beyond the treatment window, and durability of these gains remains unknown (Schachtner et al. 2025).

Riis et al. carried out a randomized, double-blind, sham-controlled crossover pilot in 22 adults with treatment-resistant depression or bipolar disorder in a current non-psychotic depressive episode; 20 participants crossed over, and 19 were used for per-protocol analysis (Riis et al. 2025). At each stimulation visit, participants completed a 1-hour MRI session with concurrent SCC sonication using a 10-minute block design (five 1-minute ON epochs interleaved with five 1-minute OFF epochs) to quantify target engagement, followed immediately by a 1-hour treatment session delivering 39–41 min cumulative real or sham stimulation across three adjacent SCC targets. Participants returned 6 days later (day 7), crossed over to the alternate condition, and the MRI and treatment sessions were repeated. Pretreatment HDRS-6 and PANAS-X were collected on days 0 and 7; PANAS-X was repeated immediately post-treatment, and HDRS-6 was repeated 24 h later (days 1 and 8) and 7 days later (days 7 and 14). Active stimulation produced a target-specific decrease in SCC BOLD activity at the group level (*p* = 0.028; usable fMRI *n*= 16) and was detectable at the individual level in 7/16 participants during the single 10-minute scan. Clinically, sadness (PANAS-X) improved immediately pre- to post-treatment, and HDRS-6 changes favored active stimulation at 24 h, with no sustained between-condition difference at 7 days. Tolerability was generally acceptable during and immediately after stimulation; however, two delayed severe psychiatric adverse events occurred following real stimulation (worsening depression with suicidal ideation, including one intentional overdose not requiring medical intervention), both resolving over the subsequent two weeks. This study built upon earlier results from the same group which reported the case of a 30-year-old woman with treatment-resistant depression who received a single 64-minute session of sonication, resulting in an immediate decrease in SCC BOLD signal on fMRI and a fall in HDRS-6 score from 11 to 0 in 24 h with sustained remission for 44 days (Riis et al. 2023). Converging evidence shows that reduction in SCC BOLD signal correlates with reduced MDD symptoms in both medical treatment and DBS (Argyelan et al. 2016).

In another case report of treatment-resistant MDD, Fan et al. 2024 delivered low-intensity ultrasound to three deep targets in a 46-year-old man with a wearable phased-array (Fan et al. 2024). Eight 5-minute pulse trains (500 kHz, 25 Hz PRF, 13% duty) were alternated on a 15-minute regimen on separate days. Sonication of the anterior nucleus of the thalamus (ANT) produced the clearest response: visual-analogue depression scores fell significantly across the two-hour session (t(2) = −8.87, *p *= 0.013), while resting-state fMRI demonstrated a reduction of default-mode network hyperconnectivity (Fan et al. 2024). In contrast, sonication of ventral capsule and bed nucleus of the stria terminalis (BNST) did not produce reductions in symptom scores.

Barksdale investigated the effects of amygdala-targeted low-intensity ultrasound in a cohort with mixed affective disorders, including MDD, anxiety, and trauma-related conditions (Barksdale et al. 2025). The study had two phases: an MR-guided target-engagement experiment (*n* = 29) in which a single 10-minute train of sonications produced an immediate 8–10% drop in amygdala BOLD signal, followed by an open-label treatment arm which repeated the protocol 15 times over three weeks. By the final visit, depressive symptoms and general-distress scores fell (HDRS-17: −5.3 ± 4.6, *p *= 0.01; MASQ: 5.7 ± 7.4, d ≈ 0.8), with parallel improvements observed on anxiety and PTSD scales. Notably, 24 non-serious adverse events were reported across 29 participants, with an even distribution of AEs across active versus sham groups. Reported adverse symptoms included decreased concentration, tingling in limbs or hands, headache, and dizziness or lightheadedness; mean reported symptom severity was 5/7. Almost all AEs resolved during the study visit, with the exception of two cases of persistent headache and one case of irritability, each of which resolved in the following days (Barksdale et al. 2025).

Attali et al. piloted a portable, navigated, metalens-based transcranial ultrasound system in five adults with treatment-resistant MDD (Attali et al. 2025). Each participant received 25 sessions (five, 5-min sessions per day for five days) targeted to the SCC. Depression severity fell rapidly: immediately post-treatment on day 5/5, mean MADRS dropped 60.9% from 37.2 ± 6.9 to 14.8 ± 8.6 by day 5 (*p* = 0.031); four of five patients met response criteria, while two achieved clinical remission. HDRS-17/−6 and QIDS-SR moved in parallel; resting-state fMRI showed increased SCC and left DLPFC connectivity, along with decreased SCC and hippocampal connectivity, potentially indicating network changes consistent with other rapid-acting antidepressant interventions. Transient sleepiness was the only common mild side effect (31/125 sessions), and post-treatment MRI was normal. However, by week 4, mean MADRS scores increased (reverted) 38.5% from post-treatment levels and stabilized at ~ 22% gains, with no patients still meeting response criteria. Maintenance dosing or booster sessions may be needed (Attali et al. 2025).

### Generalized anxiety disorder (GAD)

Mahdavi et al. 2023 (Kuhn group, UCLA) conducted an open-label pilot study investigating the effects of low-intensity transcranial focused ultrasound (tFUS) on 25 participants with treatment-resistant generalized anxiety disorder (GAD) (Mahdavi et al. 2023). The amygdala is thought to be a relevant part of pathophysiology in anxiety disorders, involved with abnormal fear and emotional processing; each participant received eight weekly 10 min sessions of low-intensity FUS directed at the right amygdala (650 kHz; 5 ms pulses at 10 Hz; duty 5%; I_SPPA_ ≈ 14.4 W cm⁻²; ISPTA ≈ 720 mW cm⁻²) (Roy et al. 2013). At post-treatment assessment, anxiety as measured by the Hamilton Anxiety Rating Scale (HAM-A) and Beck Anxiety Inventory (BAI) fell markedly (HAM-A −12.64 ± 12.51, BAI − 12.88 ± 10.42, both *p *< 0.001) and 8/25 patients (32%) entered remission (HAM-A ≤ 14) with 16/25 patients (64%) rating themselves “much” or “very much” improved on the Patient Global Impression (PGI-I) inventory. No adverse events occurred (Mahdavi et al. 2023).

Later expanding these findings into a double-blind RCT, the Kuhn group (in partnership with other UCLA investigators) has recently published interim data from 23 treatment-resistant GAD patients (anticipated *n *= 48) treated with four weeks of once-weekly ultrasound targeting the right amygdala (Spivak et al. 2025). Interim results show an improvement in anxiety in both treatment and control groups, with possible differences between groups which merit further investigation; not enough data was included in the published abstract for inclusion in our analysis. The same group has recently published a clinical trial protocol for the most ambitious effort yet to study ultrasound neuromodulation in psychiatric populations: an ongoing, double-blind, sham-controlled RCT that plans to enroll up to 100 right-handed adults with high anhedonia and moderate-to-severe MDD (Rotstein et al. 2025). It is planned that these individuals will receive three sessions of low-intensity focused ultrasound to the left caudate head across a 5–9 day window. Results have not yet been reported as of this writing.

### Schizophrenia

As of this review, Zhai and colleagues have reported the only series using FUS neuromodulation as a treatment for schizophrenia (Dong et al. 2017; Insel 2010; Zhai et al. 2023). They conducted a double-blind, randomized, sham-controlled study evaluating the efficacy of low-intensity transcranial ultrasound targeting the left DLPFC in 26 patients with predominantly negative symptoms of schizophrenia (Zhai et al. 2023). Participants were randomized (13 active, 13 sham) to 15 weekday sessions over three weeks. The Scale for the Assessment of Negative Symptoms (SANS) reflected a significant reduction in negative symptoms in the ultrasound group compared to the sham group (interaction effect: F = 16.965, *p* < 0.001, partial η² = 0.414). As the Group x Time interaction only signals that treatment trajectories diverged, post-hoc contrasts were required to pinpoint where the difference lay. Post hoc analysis revealed that the active group exhibited a substantial decrease in SANS scores (*t* = 7.397, *p* < 0.001) with no change in the sham group. Secondary outcomes, including the Positive and Negative Syndrome Scale (PANSS), also showed significant improvements in the active group. Cognitive performance on the Continuous Performance Test (CPT) improved significantly (*F* = 4.277, *p *= 0.050), though no significant effects were observed in the Trail Making Test, Symbol Coding, or Digital Span tasks. Further follow-up was not assessed. No serious adverse events were reported, though two active-group patients experienced transient difficulty falling asleep, which resolved (< 7 days). Additionally, prior motor-evoked-potential (MEP) work in a separate schizophrenia cohort using the same FUS parameters showed LTP-like increases in ipsilateral M1 excitability lasting 15 min, supporting the capacity to induce short term neuroplasticity using this ultrasound protocol (Zhai et al. 2023).

### Substance use disorder (SUD)

Mahoney et al. investigated the effects of low-intensity focused ultrasound targeting the nucleus accumbens (NAc), a target implicated in addiction and other reward disorders (e.g. OCD), in an open-label safety/feasibility pilot in four adults (*n *= 4) receiving concomitant comprehensive treatment for opioid use disorder (OUD) and polysubstance use (Peng et al. 2024; Mahoney et al. 2023). Each participant first underwent 5 min of sham sonication per hemisphere, then 20 min of active FUS to the bilateral nucleus accumbens; the first two subjects received a “lower” dose, while the latter two an “enhanced” dose. Cue-reactivity testing showed little acute effect with the low dose protocol, but in the enhanced protocol cue-induced craving for preferred drugs (e.g., heroin, alcohol, benzodiazepines) fell sharply during sonication and remained lower through 24 h post-treatment. Daily ecological-momentary-assessment over the following week confirmed significant craving reductions—for example, in Participant 3 mean opioid visual analog scale (VAS) cue-induced cravings fell from 3.6 ± 0.6 to 1.9 ± 0.4 and methamphetamine from 3.2 ± 0.4 to 0.0 ± 0.0 (all *p* < 0.01). At 90 days, maximum cue-induced craving scores were still lower than baseline for all four participants, suggesting durable benefit, though the small sample and absence of a sham-only arm limit inference. FUS was well-tolerated, produced no structural MRI changes, and only mild adverse events occurred (*n *= 10), such as transient headache, and all resolved within one day. Overall, the study provides preliminary evidence that bilateral NAc FUS is safe and may suppress substance cravings (Mahoney et al. 2023).

The same group expanded their earlier pilot into a prospective, single-arm trial of eight adults with severe OUD and multiple co-occurring substance use disorders (Rezai et al. 2025). They sonicated the bilateral NAc over four 5-minute blocks in a single session. Results were significant; measured on a patient-reported VAS 0–10 scale which quantifies cue-induced opioid craving intensity, median craving scores fell immediately (from 6.9 to 1.1 at 24 h; *p* < 0.002) and remained 91% lower at day 90 (from 6.9 to 0.6; *p *< 0.0001). Similar reductions were observed for cravings for both methamphetamine and cocaine. Urine toxicology confirmed that seven participants were abstinent at 30 days following treatment, while five remained drug-free through 90 days. Resting-state fMRI showed progressive decreases in NAc connectivity to the ventromedial prefrontal and anterior/posterior cingulate cortices over the 90-day follow-up period, suggesting dampening of reward circuit hyperconnectivity. The procedure was overall well-tolerated (9 mild headaches, 1 moderate anxiety, and no device-related serious adverse events or MRI abnormalities were noted) (Rezai et al. 2025). These results demonstrate the preliminary safety and potential durability of bilateral NAc FUS as an adjunct treatment for refractory OUD/SUD.

### Obsessive-compulsive disorder (OCD)

A published abstract from Jordan et al. reported preliminary results from a protocol exploring the effects of LIFU targeting the basal ganglia in a cohort of 21 patients with treatment-resistant obsessive-compulsive disorder (OCD). Dysfunction of the basal ganglia has previously been established as related to OCD symptom severity (Jordan et al. 2023). Initially, six participants received ultrasound targeting the caudate nucleus, but only two completed the protocol for reasons not detailed. The next fifteen participants received FUS targeting the ventral striatum; one was excluded due to travel concerns, and ten completed the protocol. Among these 10 subjects, seven individuals (70%) demonstrated a clinical response, defined as ≥ 35% reduction in Yale-Brown Obsessive-Compulsive Scale (Y-BOCS) scores or + 2 or greater movement on the Global Rating of Change (GRC). No serious adverse events were reported, though one participant withdrew due to worsening anxiety symptoms. The high dropout rate was observed primarily in the caudate nucleus group, whereas the ventral striatum appeared to be a more tolerable target. Long-term efficacy remains unclear as no further data has been published at the time of this writing (Jordan et al. 2023).

## Discussion

Neuromodulatory FUS is an innovative technology which has demonstrated early promise for the treatment of psychiatric disorders. The studies reviewed here highlight its potential in treating MDD, GAD, SUD, schizophrenia, and OCD with precision, reversibility, and minimal side effects, particularly in patients who may be refractory to conventional treatment. While the findings are promising, they also emphasize the need for standardized protocols, larger sample sizes, and long-term evaluations to fully establish the role of FUS in psychiatric care.

The evidence for FUS in major depressive disorder is the most developed among psychiatric indications. Across designs, most studies reported symptom improvement; however, controlled results were heterogeneous, with randomized trials demonstrating significant reductions in depression severity in some cohorts, while earlier pilot work showed more modest gains. Improvements in validated clinical measures such as HDRS-17, MADRS, and BDI-II scores reveal its treatment potential, though durability of effects varied widely from transient to sustained to not assessed based on the study design. The largest MDD RCT, with 30 subjects, demonstrated that the group significantly reduced HDRS-17 scores, anhedonia, cognition, and working memory, changes which all persisted or even further improved at three-month follow-up. While these are early data, they may suggest the possibility of producing a durable effect (Cheung et al. 2023). Another large MDD study found significant clinical improvement in MADRS scores, suicidal ideation, and overall mood state immediately post-treatment, which also further improved at two-week follow up (Oh et al. 2024). These early positive data emphasize the therapeutic potential of FUS for depressive disorders. Similarly, pilot studies in generalized anxiety disorder, schizophrenia, obsessive-compulsive disorder, and substance use disorder have demonstrated encouraging results, including reductions in anxiety scores, negative symptoms, and substance cravings.

The studies detailed in this scoping review targeted multiple different brain regions using a variety of sonication protocols. Further, they also reported variable outcomes, suggesting that target selection and sonication parameters need to be standardized for each indication. Protocol timing and sonication parameters are likely to be key determinants of clinical efficacy, but the present literature is insufficient to support prescriptive recommendations. Across included studies, session duration ranged from 5 to 64 min, and total course length spanned a single visit to multiple sessions delivered over up to five weeks; pulse regimes and duty cycles ranged from low‑temporal/duty averages intended to minimize heating to burst‑type paradigms with higher duty; all were within appropriate safety indices. From a patient comfort and resource perspective, fewer and shorter sessions at lower energy loads are inherently desirable; however, available data do not yet show how these choices trade off with efficacy or durability in head‑to‑head designs. Thus, protocol aggregation and standardized reporting will likely help establish convergent sonication parameter sets to better inform future studies, streamline regulatory review, enable cross‑study synthesis, and mitigate safety concerns; indeed, efforts towards this aim including the recent ITRUSST consensus are underway (Martin et al. 2024). Alternatively, the inherent flexibility in parameter space of LIFU allows for fine tuning of protocols to better optimize both treatment effect and durability of symptom improvement depending on each individual’s clinical condition and symptom profile. Unfortunately, this may prolong the process of determining a set of optimal parameters for each clinical condition, if such a set exists on a generalizable basis.

Beyond symptom scales, several studies reported neuroimaging or physiological markers consistent with target engagement and, in some cases, small‑to‑moderate effect sizes. Corroborating symptom scale changes with circuit‑level differences strengthens the biological plausibility of clinical effects and offers a compelling roadmap for future studies to follow; though serial imaging may be costly and cumbersome, it also may be necessary to assess durability of effects. Adequately powered, fMRI‑validated LIFU trials that formally link imaging changes to clinical effect sizes are a key next step.

For patients with limited therapeutic options, such as those with treatment-resistant psychiatric disorders, FUS offers a novel avenue for potential long- and/or short-term symptom relief. While the mainstay treatments of psychiatric conditions—psychotherapy and medication—typically occur on a longer-term basis, studies should investigate the potential of LIFU to induce acute changes as well. To this end, such studies might consider prespecifying brief behavioral endpoints within 24 h (such as HDRS‑6 or state‑anxiety measures) paired together with immediate post‑sonication neural correlates including fMRI changes. Acute neuromodulation might better target conditions with acute presentations, such as a substance use episode or a bout of suicidal ideation. Additionally, psychiatric conditions with limited treatment options and demonstrated impairment of normal neurocircuitry, such as post-traumatic stress disorder (PTSD) and borderline personality disorder, are potential novel indications for FUS (Iqbal et al. 2023; Reich et al. 2019). Noninvasive neuromodulation with TMS has shown efficacy in the treatment of PTSD in recent years (Petrosino et al. 2021). FUS thus holds the potential to complement this and other advanced therapies, such as deep brain stimulation (DBS), and electroconvulsive therapy (ECT), as well as pharmacological treatment, though for the time being it may prove difficult to perform direct head-to-head comparisons.

Unlike pharmacological treatments that often carry systemic side effects FUS provides localized neuromodulation with what so far appears to be a mostly favorable safety profile as evidenced by the general absence of severe adverse effects with the exception of Riis 2025. Moreover, the incisionless nature of FUS makes it a relatively minimally invasive intervention from the patient’s perspective, akin to other noninvasive brain stimulation (NIBS) techniques like transcranial magnetic stimulation (TMS). There is limited data, however, regarding the durability of FUS’s effects, which could necessitate repeated sessions akin to TMS treatments (Rizvi and Khan 2019). This opens the possibility for a chronic care model, where patients undergo regular FUS sessions to maintain therapeutic benefits. However, understanding the temporal dynamics of FUS’s effects—whether they provide long-term relief or require frequent maintenance—is a critical step for integration into clinical workflows.

Another open question surrounds expertise and scope of practice for the provision of ultrasound neuromodulation. HIFU is routinely used by neurological surgeons to irreversibly ablate brain targets. Given this, specialized expertise and training are necessary to perform such procedures. However, LIFU is used for reversible neuromodulation; though the technology is not without risks, early data indicates a promising safety profile. While LIFU is currently performed in the MRI machine, it is unclear whether the technology will evolve such that the treatment will be primarily performed without real-time MRI monitoring. This may justify its move to a TMS-like therapy with outpatient office-based treatments with neuronavigation. If LIFU no longer requires performance in an MRI scanner, it remains to be seen if neurosurgeons will continue to be the primary specialists given the small number of subspecialty practitioners and time demand of the therapy. Importantly, a significant increase in the number of patients requiring repeated treatments in the MRI scanner will put a strain on the utilization of any clinical magnet and may require substantial investments to install facilities dedicated to the procedure.

The current body of literature is hindered by several methodological limitations. Most studies reviewed involved small cohorts; larger-scale, sham-controlled randomized controlled trials are needed to prove broad-scale efficacy. Additionally, many studies fail to report long term longitudinal follow-up, which limits interpretation of evidence as the psychiatric conditions studied are chronic and may fluctuate over prolonged time periods. Future research should investigate whether repeated sessions yield cumulative benefits or diminished effectiveness over time with an emphasis on multi-year follow-up. Finally, the literature does not yet substantially explore the response to FUS in genetically diverse populations due to small cohort sizes, which raises open questions about the generalizability of findings in the setting of an increasing push for pharmacogenomic testing in psychiatry (Virelli et al. 2021).

This review itself is also subject to several limitations. By excluding animal and preclinical studies, we may have overlooked foundational mechanistic insights that could inform clinical applications. Searches were limited to three major databases, potentially excluding relevant studies published elsewhere. Additionally, there is the risk of publication bias in that it is more likely that positive studies were published. Furthermore, the heterogenous endpoints of the included studies made quantitative synthesis of studies challenging. Across the included studies, the context behind the sonication parameters was reported with inconsistency, with many not identifying whether the values represented in situ measurements, free-field conditions, or derated estimates. This variability reflects a potential broader limitation in clinical FUS literature, where heterogeneous reporting standards can hinder comparisons between studies. Moreover, because the effective acoustic dose delivered to the target can depend on a wide variety of anatomical factors, including skull density, thickness, and angle of incidence, parameter values can vary greatly between patients. The lack of consistent reporting of effect sizes and dose-response relationships prevents conclusive synthesis of parameter-dependent changes in clinical outcomes; to this end, we have summarized available data on the range of testing conditions in Table 2. Lastly, we acknowledge the small number of eligible human LIFU studies identified; this scarcity reflects the early developmental stage of clinical-focused ultrasound neuromodulation research for psychiatric indications and the need for greater exploratory clinical data. As the field grows and more trials report standardized outcomes, future reviews will be better positioned to draw stronger comparative conclusions regarding efficacy, safety profiles, and disorder-specific responses.

The commercial and regulatory landscape for FUS is still evolving. As discussed, the FDA has approved ablative MRgFUS for limited indications including medication-refractory essential tremor, tremor-dominant Parkinson’s disease and Parkinson’s dyskinesias, but psychiatric applications of FUS remain nascent (Kaplitt et al. 2024; Sridhar and Kohi 2018). There has been an uptick in startup companies seeking to bring MRI-free neuromodulatory FUS to market for a variety of indications. Recent work has seen the development of wearable ultrasonic transducers, designed for long-term neuromodulation (Tang et al. 2025). Ongoing clinical trials of novel ultrasound devices are critical to building the evidence base required for broader FDA approval, technology development, and clinician adoption in neuromodulatory FUS and other applications of the technology. For example, LIFU has also been shown to disrupt the blood-brain barrier which holds therapeutic potential for improving drug delivery in CNS cancers and other conditions (Carpentier et al. 2024). Lessons from FUS across different contexts, as well as TMS and other neuromodulatory technologies should further inform efforts to improve efficacy, scale, uptake, and reimbursement as the evidence for FUS in psychiatric disorders continues to expand.

## Conclusion

Neuromodulatory FUS has shown significant promise as an innovative therapeutic modality for psychiatric disorders. Its combination of precision, safety, and versatility offers hope for patients with few treatment options. However, addressing open questions surrounding mechanistic understanding, parameter space optimization, and durability of effects is essential for translating FUS from research to potential widespread clinical use. With continued interdisciplinary collaboration and rigorous research, FUS could meaningfully expand the treatment landscape for psychiatric conditions, offering an avenue for transformative progress in neurosurgical care.

## Supplementary Information

Below is the link to the electronic supplementary material.

Supplementary Material 1

## Abbreviations

LIFU/FUS: Low-Intensity Focused Ultrasound
HIFU: High-Intensity Focused Ultrasound
MRgFUS: Magnetic Resonance-guided Focused Ultrasound
