# Supplementary material for: Focused ultrasound neuromodulation for psychiatric disorders: a scoping review of clinical applications and current progress
Source: J Neural Transm (Vienna). 2026 Feb 19;133(7):1289–307. doi: 10.1007/s00702-026-03113-3 (PMC13428694; doi:10.1007/s00702-026-03113-3)
Supplement: Supplementary file 1 — Supplementary Material 1 [file 702_2026_3113_MOESM1_ESM.docx]

**Supplementary Table 1:** Search Terms

| PubMed | ("focused ultrasound" OR "transcranial focused ultrasound" OR "FUS" OR "ultrasound brain stimulation" OR "HIFU") AND ("neuromodulation" OR "therapy" OR "treatment" OR "stimulation") AND ("depression" OR "anxiety" OR "substance use disorders" OR "emotional wellness" OR "mood disorders" OR "addiction" OR "psychiatric disorders") |
| --- | --- |
| Embase | (("focused ultrasound" OR "high-intensity focused ultrasound" OR HIFU OR "therapeutic ultrasound" OR "MR-guided focused ultrasound" OR "magnetic resonance-guided focused ultrasound" OR MRgFUS) AND (neuromodulation OR "neural modulation" OR "brain stimulation" OR "targeted brain modulation" OR "central nervous system modulation") AND (psychology OR psychiatric OR "mental health" OR psychosis OR depression OR anxiety OR PTSD OR OCD OR schizophrenia OR "bipolar disorder" OR addiction OR "substance use disorder" OR "eating disorder" OR "behavioral disorder")) |
| Scopus | ("focused ultrasound" OR "transcranial focused ultrasound" OR "FUS" OR "ultrasound brain stimulation" OR "HIFU") AND ("neuromodulation" OR "therapy" OR "treatment" OR "stimulation") AND ("depression" OR "anxiety" OR "substance use disorders" OR "emotional wellness" OR "mood disorders" OR "addiction" OR "psychiatric disorders") |

**Supplementary Table 2: Inclusion and Exclusion Criteria**

| **Domain** | **Include** | **Exclude** |
| --- | --- | --- |
| Population | Human participants diagnosed with a psychiatric disorder ranging in severity from mild to treatment-resistant | Healthy volunteers; animal or in-vitro studies; neurological or surgical cohorts without a psychiatric diagnosis |
| Intervention | Neuromodulatory focused ultrasound (LIFU/FUS or other non-ablative protocols) applied to the brain | High-intensity focused ultrasound (HIFU), ablative MRgFUS/capsulotomy; diagnostic ultrasound; BBB-opening only; non-ultrasound neuromodulation (TMS, tDCS, ECT, etc.) |
| Outcomes | Quantitative or qualitative therapeutic or neuromodulatory outcomes (symptom scales, imaging, neurophysiology, safety, etc.) | Studies reporting only device engineering, phantom models, or no patient-level outcomes |
| Study design | Original clinical research of any design (RCTs, non-randomized trials, case series, case reports) | Narrative reviews, systematic reviews, commentaries, editorials, conference abstracts or correspondence lacking efficacy data |
| Language | English | Non-English sources without an English abstract |
| Publication status | Published, in-press, or ahead-of-print full articles; indexed conference abstracts with sufficient data | Duplicate reports, trial registrations without results, unpublished unindexed data |
| Timeframe | No date restriction up to final search (May 2025) |  |
